# Single-center retrospective analysis of 454 culture-positive patients with tinea capitis and measurement of pathogens regarding thermal tolerance at 37°C

**DOI:** 10.3389/fmed.2025.1550270

**Published:** 2025-08-25

**Authors:** Xiujiao Xia, Jiajia Li, Huilin Zhi, Zehu Liu

**Affiliations:** Department of Dermatology, Hangzhou Third People’s Hospital, Hangzhou Third Hospital Affiliated to Zhejiang Chinese Medical University, Hangzhou, China

**Keywords:** tinea capitis, epidemiology, Hangzhou, thermal tolerance, dermatophyte

## Abstract

**Background:**

This study aimed to analyze the epidemiological characteristics of tinea capitis (TC) and the changing trend of the pathogenic fungal spectrum in Hangzhou to assess the thermal tolerance of these pathogenic dermatophytes at 37°C.

**Methods:**

Clinical, demographic, and mycological data of 454 TC patients were retrospectively collected.

**Results:**

Among children with TC, 198 were females and 201 were males, with a median age of 5 years. Among adults, there were 44 patients with TC among females and 11 patients with TC among males with a median age of 65 years. *Microsporum canis* was the most common pathogen (311, 68.50%), followed by *Trichophyton violaceum* (48, 10.57%), *T. tonsurans* (35, 7.71%), and *T. mentagrophytes* complex (30, 6.61%). The proportion of *M. canis* in the children group and adult group was 73.43% and 32.73%, respectively. After subculture, visible growth at 25°C were observed in 381 isolates, and 351 grew at 37°C. Compared to the proportion growing at 37°C, the proportion growing at 25°C was significantly greater for isolates from gray patch, *M. canis,* and *T. mentagrophytes* complex. There was no significant difference in the proportion growing at the two temperatures for other isolates, and all 10 strains of *Nannizzia gypsea* grew well at 37°C.

**Conclusion:**

*Microsporum canis* is the dominant pathogen of TC. Elderly women (postmenopausal women) were the main affected group of adult TC, and the proportion of anthropophilic dermatophytes in the adult group was significantly higher than that in the children group. Most dermatophytes were well tolerated at 37°C, particularly *N. gypsea*.

## 1 Introduction

Tinea capitis (TC) is a dermatophyte infection affecting hairs and scalps, which mainly occurs in preadolescent children but sometimes affects adults (1). The clinical manifestations of this superficial skin infection vary from asymptomatic carriers to kerion with intense inflammation leading to permanent scar (2). TC is prevalent worldwide, and its prevalence varies geographically and demographically. The prevalence of TC among superficial fungal diseases varies widely worldwide, ranging from 1 to 68.3% (3–6). These data should be interpreted with caution given the heterogeneity of diseases included in various studies. TC is one of the most common health problems due to its high incidence among preadolescent children (1). China has a vast territory and a large population, thus different regions of China have different natural and social environments. These factors determine the epidemiological characteristics of TC in different regions of China. In the central region of China, anthropophilic dermatophytes are the main pathogens, while in other regions, the pathogens are mainly zoophilic dermatophytes, particularly *M. canis* (7). Hangzhou is located in the southeast coast of China, where the level of economic and social development is higher compared to the northwest region. Previous reports have shown that the pathogenic spectrum of TC in Hangzhou has undergone marked changes over the past decade. Before 2011, anthropophilic dermatophytes (mainly *T. violaceum*) were the dominant pathogens in Hangzhou and now zoophilic dermatophytes (particularly *M. canis*) are the dominant pathogens (8, 9).

Dermatophytes are a group of fungi that are morphologically, physiologically, and antigenically closely related. Dermatophytes are keratinophilic fungi causing infections of the skin, nails, and hair of mammals and feathers of birds. These fungi are the most common pathogenic fungi for humans. The optimum growth temperature for this type of fungi is 22–28°C; thus, room temperature is used as the primary isolation temperature for dermatophytes. The results showed that the pathogenicity of fungi was positively correlated with 37°C tolerance (10), suggesting that 37°C tolerant fungi can severely infect humans.

Based on previous studies (8, 9), we re-analyzed patients with TC in our center in the past 5 years to understand the epidemiological characteristics of TC and the changing trend of pathogenic fungal spectrum in the Hangzhou area in the past 13 years. We initially explored the tolerance of the pathogenic fungi at 37°C.

## 2 Materials and methods

From October 2019 to April 2024, we identified TC patients at our hospital by reviewing the database of the Laboratory Information System of the Mycology Laboratory and Medical Information System. Patients with TC were recruited for analysis only when they fulfilled the following criteria: (a) microscopic true hair invasion in the form of ectothrix or endothrix; and (b) culture-proven dermatophyte infection of the scalp. Exclusion criteria were as follows: (a) revisiting patients, or (b) microscopically positive but being culture-negative. General information of patients included sample number, name, gender, age, geographical localization, duration of the disease until confirming the diagnosis, history of animal contact, and clinical patterns at the time of the first visit. Clinical patterns were classified as gray patch, black dot-type, kerion, and favus. Patients less than 18 years old were grouped as “Children” and those older than 18 years were grouped as “Adults.” Prescriptions were recorded. Antifungal treatment options were categorized into five groups: oral itraconazole (ITZ), oral terbinafine (TRB), topical antifungal, oral antifungal combined with topical antifungal agents, oral antifungal agents combined with corticosteroids, other, and none.

Mycological examinations, including direct microscopy and fungal culture, were conducted in the mycology laboratory of our hospital. Broken hairs were taken with a pair of forceps and scales from the scalp were collected by scrapping lesions with a sterile lancet. Samples used for microscopy examination were put on glass slides and treated with a fungal fluorescence staining solution (Anhui Delaikang Biological Medical Technology Co., Ltd.; China). Samples were also inoculated into Sabouraud dextrose agar after adding cycloheximide and chloramphenicol (Hangzhou Bin He Microorganism Reagent. Co., Ltd.; China). Incubation at 25 °C was conducted for at least 10 days. Cultures with no obvious fungal growth were kept for 3 weeks before being judged negative. The identification of individual strains was based on the macroscopic and microscopic features of the colonies. The isolates that could not be unequivocally identified using the conventional methods were subjected to molecular identification by amplification and sequencing of internal transcribed spacers (ITS) of ribosomal DNA with primers ITS1 and ITS4. The sequencing results were compared with those deposited in the GenBank.

Once the isolate was identified as dermatophytic fungus of TC, the isolate was subcultured to two SDA slants and, respectively, incubated at 25 °C and 37 °C for 3 weeks. The growth was continuously monitored. The growth was considered positive when a colony was visible without magnification. These groups were compared for thermal tolerance at 2 temperatures, 25 °C and 37 °C, reflecting ambient and mammalian temperatures, respectively.

All data were visualized using R (version 4.3.2) and GraphPad Prism (version 8.0.1) software. Differences in gender, clinical patterns, and pathogens between children and adults, and isolate growth were compared using Fisher’s exact test. For growth proportions, 95% confidence intervals (CIs) were calculated using the Wilson method to indicate the precision of the estimates. *p* values were considered significant at the 5% level. The study was conducted in accordance with the Declaration of Helsinki, and approved by the Medical Ethics Committee at the Hangzhou Third People’s Hospital (protocol code: 2024KA146, date of approval: 2024-11-19).

## 3 Results

**Figure 1:**
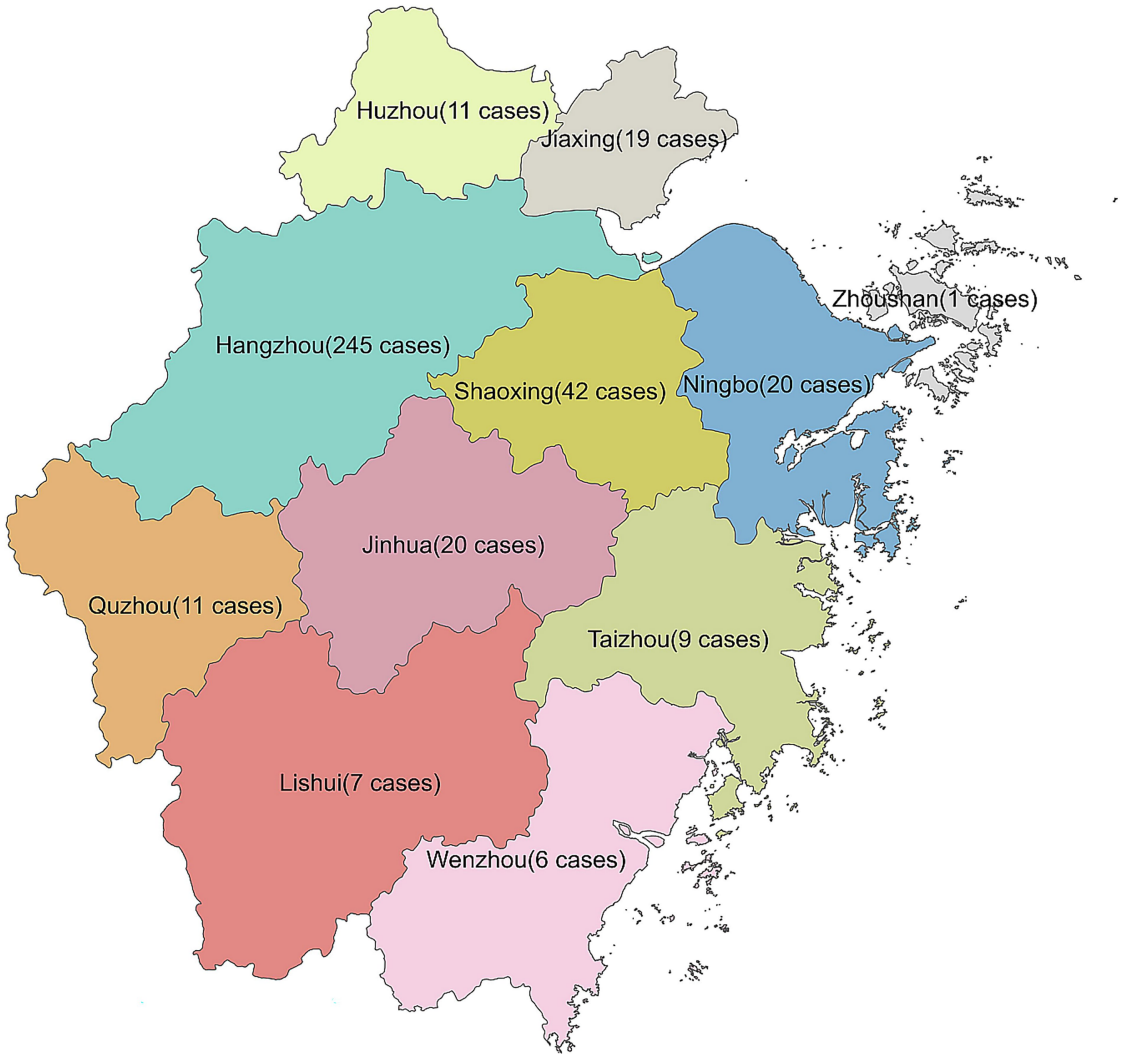
Geographical distribution of 391 patients with TC from the Zhejiang province of China.

**Figure 2:**
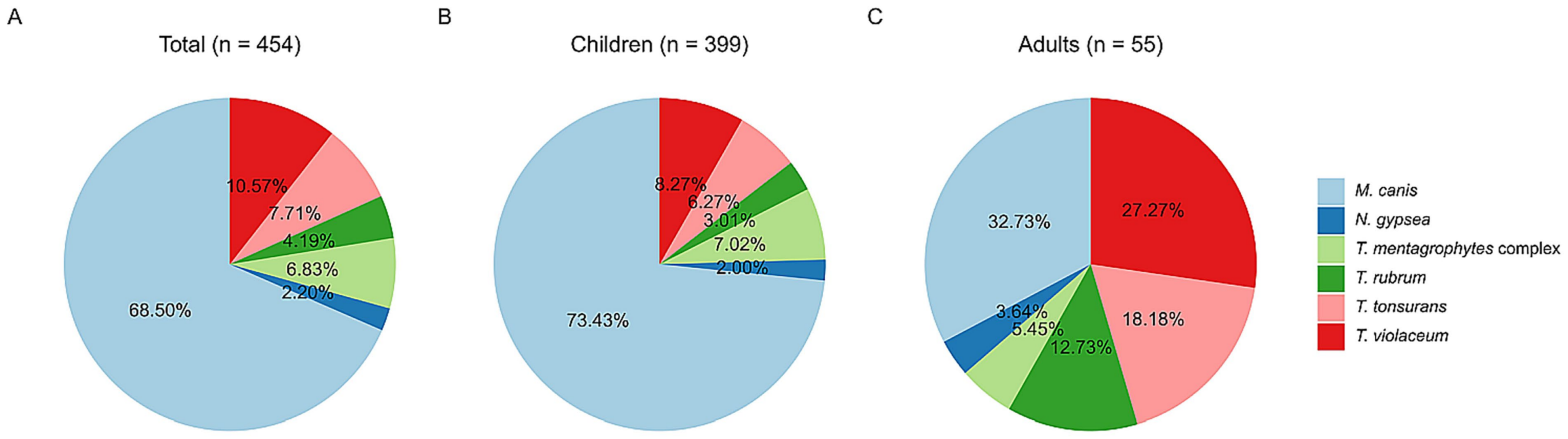
Frequency distribution of culture results of pathogens. Overall distribution **(A)**, pediatric group **(B)**, adult group **(C)**.

We included 454 patients with culture-positive tinea capitis (Table 1). There were 212 males and 242 females whose ages ranged from 1 month to 89 years, with a median age of 6 years. The duration of the disease ranged from 1 day to 30 years, with a median duration of 60 days. Of 454 cases, 63 cases were not from Zhejiang Province, and 391 were from Zhejiang Province. Among those from Zhejiang province, 245 cases (62.66%%) were from Hangzhou, 42 were from Shaoxing, 20 were from Ningbo and Jinhua, 19 were from Jiaxing, 11 were from Quzhou, and 11 were from Huzhou. Fewer cases were from other areas (Figure 1). Gray patch was the most common lesion (282, 62.11%), followed by kerion (99, 21.81%), black dot (72, 15.86%) and favus (1, 0.22%). Five strains were identified using ITS sequencing, including 2 *T. mentagrophytes* complex (GenBank accession number: PX063752, GenBank accession number: PX063765), 1 *T. rubrum* (GenBank accession number: OM919545), 1 *M. canis* (GenBank accession number: PX063767) and *T. schoenleinii* (GenBank accession number: PX063660). The remaining strains were identified to species level based on morphological characteristics. Among 454 positive cultures, there were 311 (68.50%) *M. canis*, 48 (10.57%) *T. violaceum*, 35 (7.71%) *T. tonsurans*, 30 (6.61%) *T. mentagrophytes* complex, 19 (4.19%) *T. rubrum*, 10 (2.20%) *N. gypsea,* and 1 *T. schoenleinii* (Figure 2A). One-hundred eighty-nine (41.63%) patients had close contact with animals, including cats (135, 71.42%), rabbits (25, 13.23%), dogs (21, 11.11%), and hamsters (8, 4. 24%).

**Table 1:** Demographics, clinical characteristics, and pathogens of patients with TC.

Characteristics	Child patients, *n* (%)	Adult patients, *n* (%)	*p* value
Total cases	399	55	NA
Median age, years (range)	5 (0.083–15)	65 (18–89)	NA
Female	198 (49.62)	44 (80.00)	<0.0001^*^
Disease course, days (range)	30 (1–730)	90 (7–10,950)	NA
Clinical patterns			
Gray patches	267 (66.92)	15 (27.27)	<0.0001^*^
Kerions	80 (20.05)	19 (34.55)	0.0224^*^
Black dots	52 (13.03)	20 (36.36)	<0.0001^*^
Pathogen			
*M. canis*	293 (73.43)	18 (32.73)	<0.0001^*^
*T. violaceum*	33 (8.27)	15 (27.27)	0.0001^*^
*T. mentagrophytes* complex	28 (7.02)	3 (5.45)	>0.9999
*T. tonsurans*	25 (6.27)	10 (18.18)	0.005^*^
*T. rubrum*	12 (3.01)	7 (12.73)	0.0042^*^
*N. gypsea*	8 (2.00)	2 (3.64)	0.3466

NA, not applicable. ^*^p < 0.05 was considered statistically significant.

**Figure 3:**
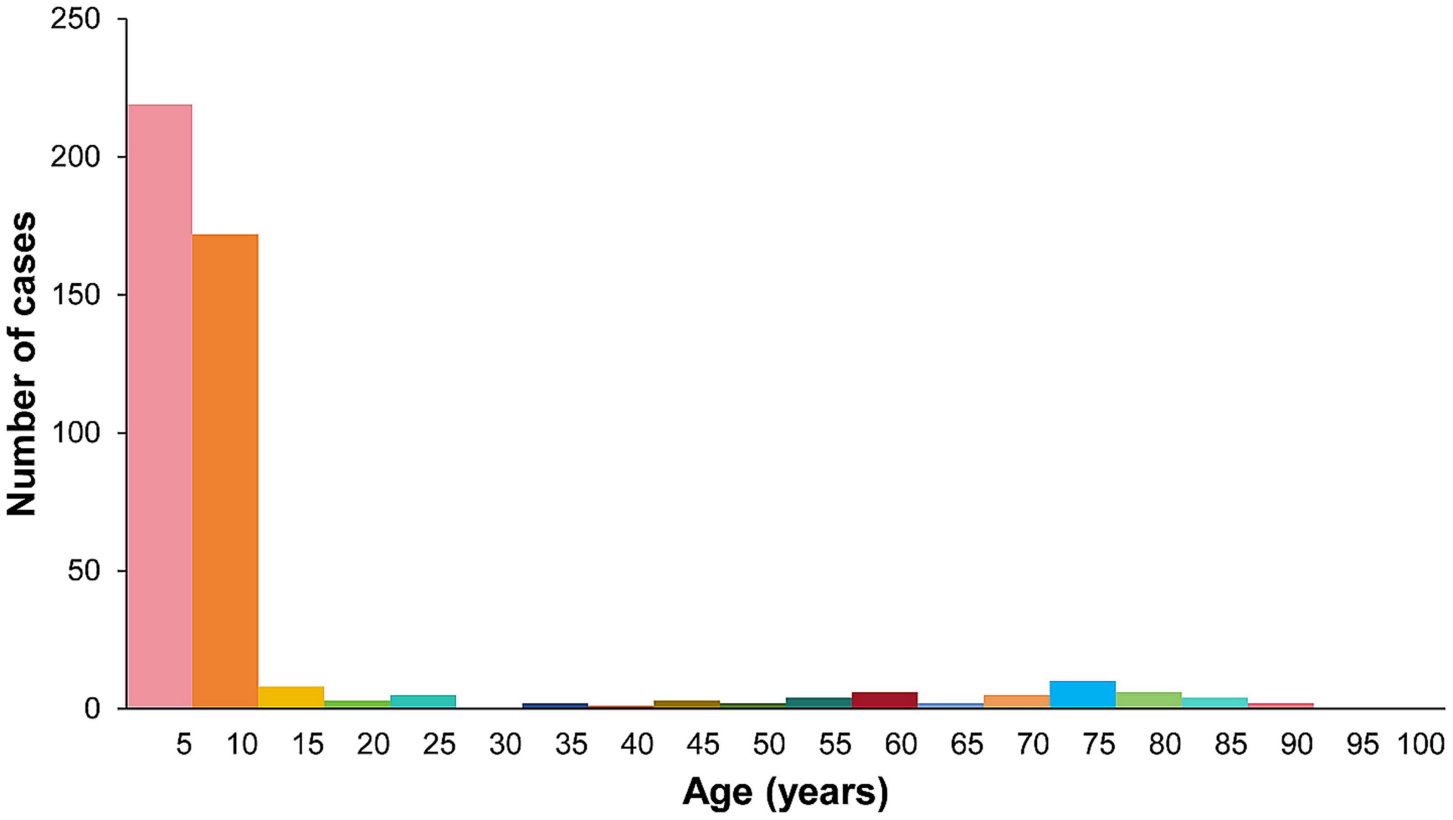
Age distribution of patients with TC.

**Figure 4:**
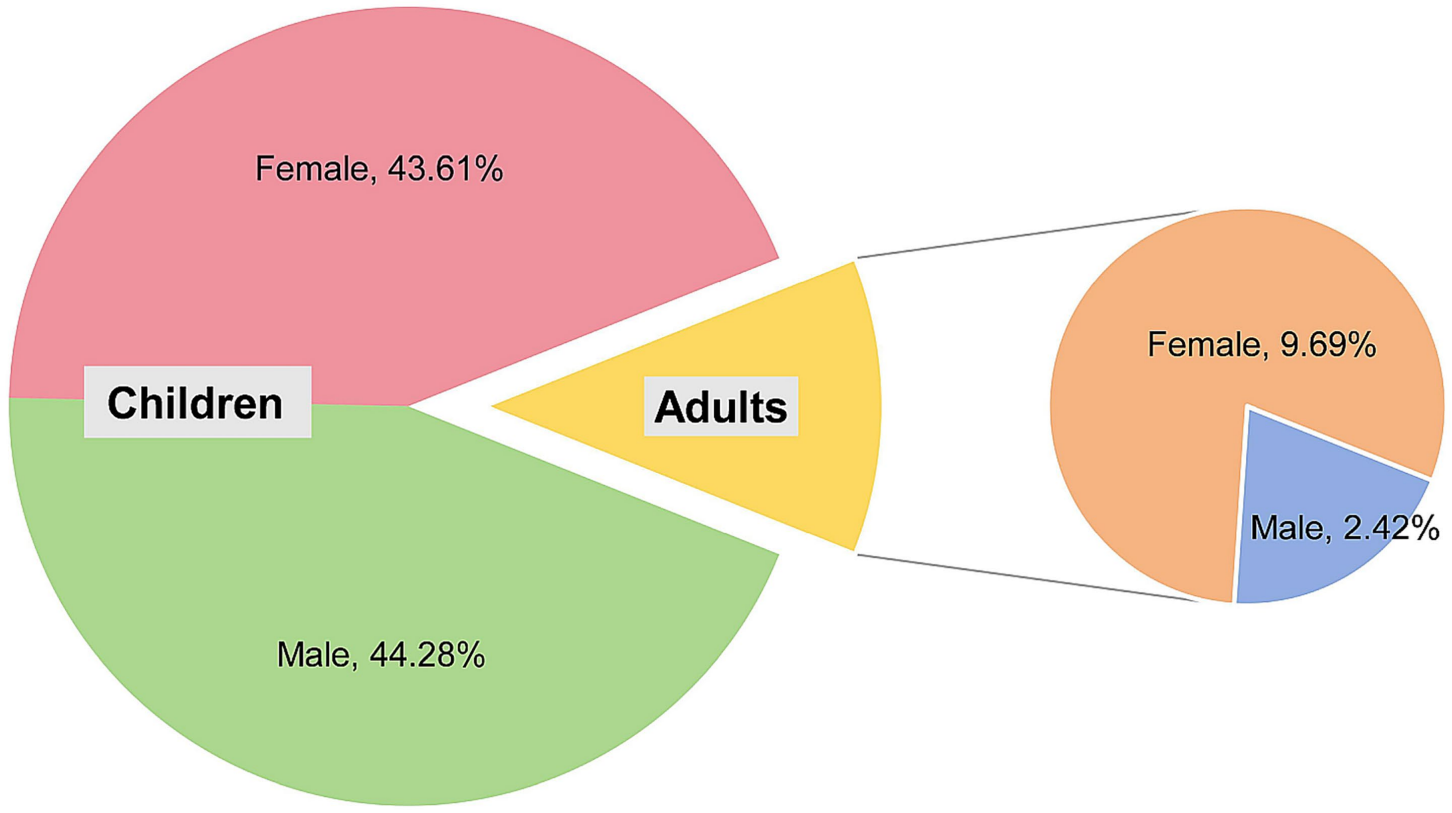
Gender distribution of patients with TC.

There were 399 cases (87.89%) in the children group (Figure 3), including 201 males and 198 females (Figure 4). Their median age was 5 years (0.083–15 y) and the median duration was 30 days (1–730 d). Of 399 children with TC, 267 cases (66.92%) presented with gray patches, 80 (20.05%) with kerions, and 52 (13.03%) with black dots. *M. canis* (293, 73.43%) was the dominant causative fungus, followed by *T. violaceum* (33, 8.27%), *T. mentagrophytes* complex (28, 7.02%), *T. tonsurans* (25, 6.27%), *T. rubrum* (12, 3.01%), and *N. gypsea* (8, 2.00%) (Figure 2B).

Of 55 adult patients with tinea capitis, 44 were females and 11 were males (Figure 4), with a median age of 65 years (18–89 y). The percentage of females was significantly higher than that of males (male:female ratio of 1:4.4, *p* < 0.0001). Among female patients, 42 patients (95.45%) were in the postmenopausal stage. The median duration of the disease until confirmation of diagnosis was 90 days (7–10,950 d). Black dots (20, 36.36%) were the most common type, followed by kerions (19, 34.55), gray patches (15, 27.27%), and favus (1, 1.82%). Similarly, the most common pathogen among adults with TC was also *M. canis* (18, 32.73%), followed by *T. violaceum* (15, 27.27%), *T. tonsurans* (10, 18.18%), *T. rubrum* (7, 12.73%), etc. (Figure 2C).

Data from the Medical Information System could only show the prescription patterns, not the therapeutic responses. Among 454 patients, 177 patients (38.99%) were treated with TRB, 100 (22.03%) patients were treated with ITZ, 96 (21.15%) patients were treated with topical antifungal agents, 23 (5.07%) patients were treated with oral antifungal agents combined with corticosteroids (mainly in kerions), 13 (2.86%) patients were treated with oral antifungal agents combined with topical antifungal agents, and 45 patients did not receive antifungal agents or were lost to follow-up.

**Figure 5:**
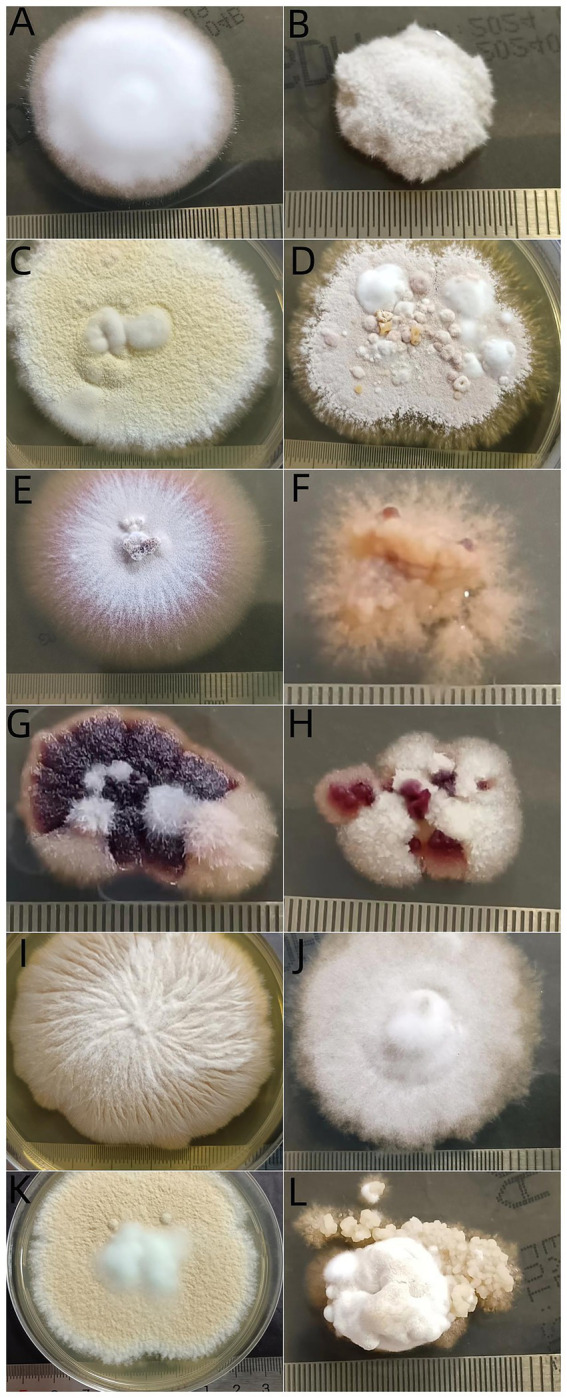
Morphogenesis of dermatophyte isolates after incubation at 2 temperatures for 21 days. *Trichophyton rubrum* at 25°C **(A)** and at 37°C **(B)**, *T. mentagrophytes* complex at 25°C **(C)** and at 37°C **(D)**, *T. tonsurans* at 25°C **(E)** and at 37°C **(F)**, *T. violaceum* at 25°C **(G)** and at 37°C **(H)**, *M. canis* at 25°C **(I)** and at 37°C **(J)**, *N. gypsea* at 25°C **(K)** and at 37°C **(L)**.

During a three-week subculture for thermal tolerance test, visible growths at 25°C and at 37°C were observed in 381 (83.92%) and 351 isolates (77.31%), respectively, suggesting that there was a significant difference in growth ratio between the two temperatures (Table 2). In terms of clinical type, among 282 isolates from gray patches, 229 (81.21%) grew at 37°C and 247 (87.59%) grew at 25°C. There was a significant difference between the two temperatures (P<0.05), and isolates from other types of TC showed no difference in growth ratio at these temperatures. Regarding the different species, all *N. gypsea* isolates grew at 37°C. The proportions growing at 37°C were significantly lower for isolates of *T. mentagrophytes* complex (25, 83.33%) and *M. canis* (251, 80.71%) than isolates of *T. mentagrophytes* complex (28, 93.33%) and *M. canis* (274, 88.10%) at 25°C (*p* < 0.05). There was no difference in the proportion of the remaining isolates growing at these two temperatures. Interestingly, the morphology of fungal colonies cultured at 37°C was remarkably different from that cultured at 25°C. Most colonies cultured at 37°C had fewer conidia and aerial mycelia (Figure 5).

**Table 2:** Growth tolerances for isolates from TC at 2 temperatures.

Origin, Pathogen	Isolate growth, *n* (% [95%CI])				*p* value
	At 25°C		At 37°C		
	Yes	No	Yes	No	
Origin					
Total	381 (83.92 [80.26–87.01])	73 (16.08 [12.99–19.74])	351 (77.31 [73.24–80.93])	103 (22.69 [19.07–26.76])	0.0148^*^
Gray patches	247 (87.59 [83.23–90.94])	35 (12.41 [9.06–16.77])	229 (81.21 [76.24–85.33])	53 (18.79 [14.67–23.76])	0.0481^*^
Kerions	84 (84.85 [76.50–90.60])	15 (15.15 [9.40–23.50])	77 (77.78 [68.64–84.84])	22 (22.22 [15.16–31.36])	0.2739
Black dots	49 (68.06 [56.61–77.67])	23 (31.94 [22.33–43.39])	44 (61.11 [49.56–71.53])	28 (38.89 [28.47–50.44])	0.486
Favus	1 (100 [20.65–100])	0 (0 [0–79.35])	1 (100 [20.65–100])	0 (0 [0–79.35])	>0.9999
Pathogen					
*M. canis*	274 (88.10 [84.03–91.24])	37 (11.90 [8.76–15.97])	251 (80.71 [75.96–84.71])	60 (19.29 [15.29–24.04])	0.0147^*^
*T. violaceum*	39 (81.25 [68.06–89.81])	9 (18.75 [10.19–31.94])	37 (77.08 [63.46–86.69])	11 (22.92 [13.31–36.54])	0.8021
*T. mentagrophytes* complex	28 (93.33 [78.68–98.15])	2 (6.67 [1.85–21.32])	25 (83.33 [66.44–92.66])	5 (16.67 [7.34–33.56])	0.0014^*^
*T. tonsurans*	20 (57.14 [40.86–72.02])	15 (42.86 [27.98–59.14])	18 (51.43 [35.57–67.01])	17 (48.57 [32.99–64.43])	0.8106
*N. gypsea*	10 (100 [72.25–100])	0 (0 [0–27.75])	10 (100 [72.25–100])	0 (0 [0–27.75])	>0.9999
*T. rubrum*	9 (47.37 [27.33–68.29])	10 (52.63 [31.71–72.67])	9 (47.37 [27.33–68.29])	10 (52.63 [31.71–72.67])	>0.9999
*T. schoenleini*	1 (100 [20.65–100])	0 (0 [0–79.35])	1 (100 [20.65–100])	0 (0 [0–79.35])	>0.9999

CI, confidence interval. ^*^p < 0.05 was considered statistically significant.

## 4 Discussion

TC can affect individuals of any age (11). Interestingly, none of the patients in our report were in the 15–18 age group. The prevalence was generally lower in adults than in children because of the increased presence of fungistatic saturated fatty acids in the sebum of adolescents (12). The results showed that the age distribution of TC was L-shaped unimodal (Figure 3), suggesting the presence of significantly more pediatric patients than adult patients. Surprisingly, a study from Taiwan showed that TC was significantly more frequent in adults than in children, showing a bimodal distribution pattern (13). This suggests that there may be regional differences in the distribution of TC with respect to age.

Short hair facilitates the access of spores to the scalp and increases the transmission (12), thus, boys are at a greater risk of TC than girls. Consistently, many reports, including those from China (7, 11, 14), confirmed these findings (2, 15–17). However, female predominance in childhood TC was reported by authors from different regions (13, 18, 19). Different from most of the previous reports, the number of male and female cases in the children group was similar (201 vs. 198). Therefore, the gender ratio among pediatric cases may vary in different countries (13). Figure 4 shows that the gender distribution in the adult and child groups was quite different. The percentage of females was significantly higher among adults with a male/female ratio of 1:4.4. A female predominance among adults with TC has been reported worldwide. This widespread phenomenon is attributed to the involution of sebaceous glands due to decreased blood estrogen levels after menopause (20, 21).

The pathogenic organism of TC varies in different geographical regions and time periods (21). *T. tonsurans* is the predominant agent of TC in North America and Caribbean countries (12, 22–24). In contrast, *T. violaceum* and *T. soudanense* are the main causative fungal species for TC in Africa (25–28). In some European and Asian countries, including Japan and Korea, *M canis* is the primary pathogen for TC (29, 30). Consistent with previous studies from Chinese Mainland (7), the most common pathogen was zoophilic *M. canis* (68.50%) in this study, followed by anthropophilic *T. violaceum* (10.57%).

Socioeconomic factors also play an important role in the spontaneous evolution of fungal distribution (11). Even, the dominant pathogens are different in different regions of the same country. Zoophilic fungus *M. canis* is prominent in developed regions, such as Guangzhou, Shanghai, and Beijing, with wages being higher and pets being more popular, while anthropophilic *T. violaceum* is the dominant pathogen in developing regions, such as central China, due to the high population density that leads to overcrowding, poor socioeconomic status, and poor hygiene (7, 31). Similarly, *M. canis* is the most common agent in Mexico, except for the city Monterrey, where *T. tonsurans* is the dominant pathogen (32).

Although *M. canis* was also the most common pathogen among adults, the pathogen spectrum of tinea capitis was significantly different between adults (*M. canis* accounted for 32.73%) and children (*M. canis* accounted for 73.43%) (Figure 2). The dominance of zoophilic pathogens in children may be attributed to the higher incidence of animal contact among them. Contact with cats and dogs may lead to infection *with M. canis* (33). Transmission of anthropophilic dermatophytes may occur through concurrent tinea infection in other sites of the body (13). More than half of adult patients with TC had concurrent tinea infection on other skin sites. The pathogenesis of TC among adults may be direct transmission from pre-existing dermatophytosis from other skin sites and infection with anthropophilic agents, such as *T. rubrum* and *T. tonsurans* (34). The increasing prevalence of TC in adults could be attributed to factors such as population aging, immune system alterations caused by systemic conditions (e.g., diabetes and malignancies), and the administration of immunosuppressive drugs (34). Additionally, consistent with previous reports, the median duration of TC among adults was much higher than that among children (90 days vs. 30 days) in our study (34, 35). Delayed diagnosis may be due to polymorphic and atypical clinical manifestations of adult TC (36).

**Figure 6:**
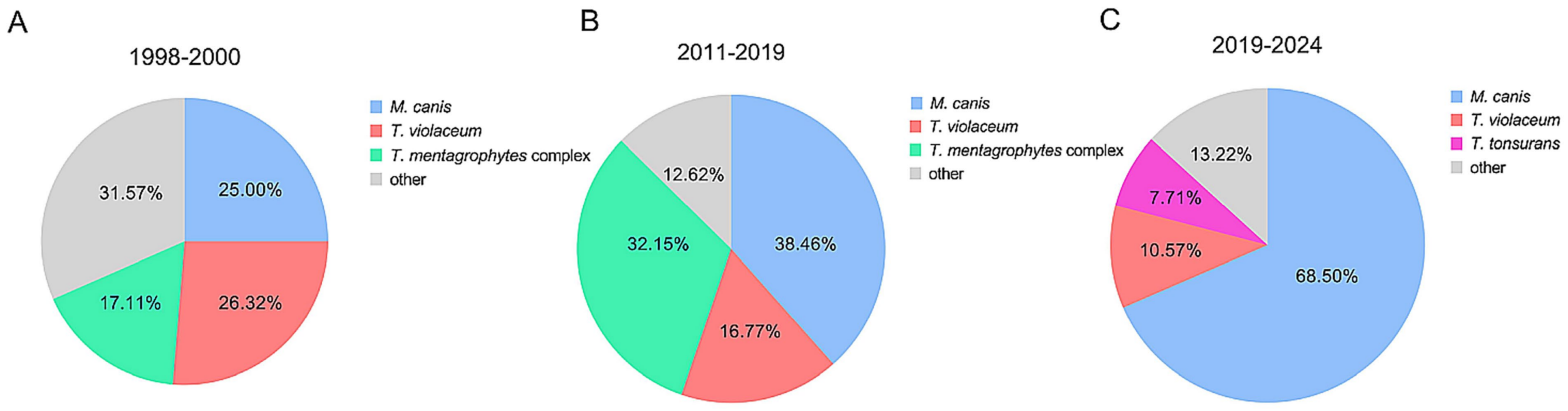
Distribution of pathogens among culture results according to the time period [previous data were obtained from Xia et al. and Zhi et al. (8, 9)]. 1998–2000 **(A)**, 2011–2019 **(B)**, and 2019–2024 **(C)**.

With the rapid economic development and the changes in living habits, the pathogen spectrum of TC in China has undergone great changes in the past 60 years. After 1985, the predominant pathogens of TC gradually shifted from anthropophilic pathogens to zoophilic pathogens (37). Our center conducted two retrospective analyses of tinea capitis, from 1998 to 2000 and from 2011 to 2024 (8, 9). Figure 6 shows that *T. violaceum* (26.32%) and *M. canis* (25.00%) were the leading causative fungus, followed by *T. mentagrophytes* complex (17.11%) between 1998 and 2000. Between 2011 and 2019, *M. canis* (39.5%) was the most frequent dermatophyte followed by *T. mentagrophytes* complex (32.2%) and *T. violaceum* (16.8%), but in the present study, the proportion of *M. canis* (68.50%) was the highest, suggesting that infections due to zoophilic agents (mainly *M. canis*) have increasingly occurred in recent years. These findings were similar to those of previous reports (7, 12, 25, 38). It should be mentioned that the main reservoirs of *M. canis* are cats and dogs (33), while rabbits are the main reservoirs of *T. mentagrophytes* complex (39).

To infect humans, fungi must meet four criteria: growth at human body temperature, circumvention or penetration into surface barriers, dissolution and absorption of tissues, and resistance to immune defenses, including high body temperature (40). Different from superficial, non-inflammatory fungal infection, kerion is an inflammatory type of TC, which is caused by a T cell-mediated hypersensitivity reaction in response to causative dermatophytes (41), particularly zoophilic and geophilic ectothrix dermatophytes, such as *M. canis*, *T. mentagrophytes* complex and *N. gypsea* (42). These zoophilic species and the geophilic organism *N. gypsea* are also the main organisms causing “Gray patch” TC. “Black dot” TC is mainly caused by anthropophilic dermatophytes such as *T. tonsurans*, *T. soudanense*, *T. violaceum,* and *M. audouinii* (43). Based on the limited data, our study showed that most isolates of dermatophytes grew well at 37°C, although not as well as 25°C. Clinically, strains isolated from kerion and black dot TC showed comparable growth at 37°C versus 25°C, whereas those from gray patch TC exhibited moderately reduced growth at 37°C. Mycologically, *M. canis* and *T. mentagrophytes* complex demonstrated slightly impaired growth at 37°C, while other pathogens maintained good thermal tolerance, particularly *N. gypsea*. These observations suggest that 37°C tolerance in dermatophytes may represent an active adaptive mechanism. Notably in kerion cases, beyond thermal adaptation, multiple additional factors likely contribute to the infectivity of zoophilic and geophilic dermatophytes. Thermal tolerance is necessary but not sufficient for microorganism invasion (44). Casadevall and colleagues found that fungi from insects and mammals had greater thermal tolerances compared to isolates from soils and plants (44). They found that most isolates grew well between 12°Cand 30°C, but with every 1° increase in temperature >30°C, around 6% fewer strains could grow (44). Morphogenesis is another important virulence factor associated with fungal movement (40). We found that most colonies cultured at 37°C had fewer conidia and aerial mycelia than those cultured at 25°C. This result might have originated from the environmental adaptive autonomous selection of fungi. The growth of round or oval cells is an essential characteristic of fungi (45).

Clinical evidence indicates that antifungal treatment must be guided by the identified pathogen species (46–51). In general, systemic antifungals demonstrate significantly greater efficacy against endothrix infections (e.g., those caused by *Trichophyton* spp.) compared to ectothrix infections (e.g., those caused by *Microsporum canis*). TRB is recommended as the first-line antifungal agent for *Trichophyton* spp. infections, while ITZ or griseofulvin should be preferred for *Microsporum*/*Nannizzia* spp. infections (43). In our study, the proportion of cases treated with TRB was comparable to those receiving ITZ, despite the high prevalence of *M. canis* (68.50%). Further controlled clinical trials are needed to thoroughly assess the comparative efficacy of these antifungal regimens.

This study was conducted at one hospital and had access to a limited amount of data, which may lead to failure in demonstrating the differences in clinical findings. In particular, the results of the thermal tolerance test were absent. However, the results of our epidemiological survey on TC were consistent with the results of national reports (7). This temperature tolerance test is only a preliminary study. In the future, we will continue to conduct thermal tolerance studies for dermatophytes, using petri dishes as the culture medium to quantitatively assess growth under gradient incubation temperatures.

## Data Availability

The original contributions presented in the study are included in the article/supplementary material, further inquiries can be directed to the corresponding authors.
